# What matters and influence resuscitation preference? Development, field testing, and structural validation of items among older patients in the emergency department

**DOI:** 10.1186/s12877-022-03707-y

**Published:** 2022-12-23

**Authors:** Stine Hanson, Søren Kabell Nissen, Dorthe Nielsen, Annmarie Lassen, Mikkel Brabrand, Roberto Forero, Jens Søndergaard Jensen, Jesper Ryg

**Affiliations:** 1 Department of Regional Health Research, Center-Esbjerg, University of Southern Denmark, Finsensgade 35, 6700 Esbjerg, Denmark; 2 Center for Global Health, Migrant Health Clinic, Odense University Hospital, University of Southern Denmark, J.B. Winsløws Vej 19, 5000 Odense, Denmark; 3 Geriatric Research Unit, Department of Geriatric Medicine, Odense University Hospital, Odense, Denmark; 4 Department of Clinical Research, University of Southern Denmark, J.B. Winsløws Vej 19, 5000 Odense, Denmark; 5 Department of Emergency Medicine, Odense University Hospital, Odense, Denmark; 6 Institute of Clinical Research, University of Southern Denmark, J.B. Winsløws Vej 19, 5000 Odense, Denmark; 7 Department of Emergency, Medicine, Hospital of South-west Jutland, Esbjerg, Denmark; 8 University of Southern Denmark, Institute of Regional Health Research, Center-Esbjerg, University of Southern Denmark, Finsensgade 35, 6700 Esbjerg, Denmark; 9 Simpson Centre for Health Services Research, University of NSW, Liverpool BC,, NSW 1871 Australia; 10 Ingham Institute for Applied Medical Research, Liverpool Hospital, Liverpool BC, NSW 1871 Australia; 11 The Research Clinic for Functional Disorders and psychosomatics, Aarhus University Hospital. Nordre Ringgade, 1,8000 Aarhus, Denmark

**Keywords:** Resuscitation, Preferences, End of life, Emergency department, Independence, Bifactor analysis

## Abstract

**Background:**

Decisions about resuscitation preference is an essential part of patient-centered care but a prerequisite is having an idea about which questions to ask and understand how such questions may be clustered in dimensions. The European Resuscitation Council Guidelines 2021 encourages resuscitation shared decision making in emergency care treatment plans and needs and experiences of people approaching end-of-life have been characterized within the physical, psychological, social, and spiritual dimensions. We aimed to develop, test, and validate the dimensionality of items that may influence resuscitation preference in older Emergency Department (ED) patients.

**Methods:**

A 36-item questionnaire was designed based on qualitative interviews exploring what matters and what may influence resuscitation preference and existing literature. Items were organized in physical, psychological, social, and spiritual dimensions. Initial pilot-testing to assess content validity included ten older community-dwelling persons. Field-testing, confirmatory factor analysis and post-hoc bifactor analysis was performed on 269 older ED patients. Several model fit indexes and reliability coefficients (explained common variance (ECV) and omega values) were computed to evaluate structural validity, dimensionality, and model-based reliability.

**Results:**

Items were reduced from 36 to 26 in field testing. Items concerning religious beliefs from the spiritual dimension were misunderstood and deemed unimportant by older ED patients. Remaining items concerned physical functioning in daily living, coping, self-control in life, optimism, overall mood, quality of life and social participation in life. Confirmatory factor analysis displayed poor fit, whereas post-hoc bifactor analysis displayed satisfactory goodness of fit (χ^2^ =562.335 (*p*<0.001); root mean square error of approximation=0.063 (90% CI [0.055;0.070])). The self-assessed independence may be the bifactor explaining *what matters* to older ED patients’ resuscitation preference.

**Conclusions:**

We developed a questionnaire and investigated the dimensionality of what matters and may influence resuscitation preference among older ED patients. We could not confirm a spiritual dimension. Also, in bifactor analysis the expected dimensions were overruled by an overall explanatory general factor suggesting independence to be of particular importance for clinicians practicing resuscitation discussions in EDs. Studies to investigate how independence may relate to patients’ choice of resuscitation preference are needed.

**Supplementary Information:**

The online version contains supplementary material available at 10.1186/s12877-022-03707-y.

## Introduction

Shared decision making and the question “What matters to you?” is important for the practice of patient-centered care [1]. This entails understanding what matters to the patient before weighing treatment options against values and preferences [2, 3]. Conversations about resuscitation options is often the preferred subject for shared decision making among patients in the hospital setting and therefore resuscitation shared decision making with older patients should be prioritized by clinicians [4, 5]. Here, the Emergency Department (ED) setting in particular has been proposed as a “teachable moment” for patients in conversations about goals-of-care [6]. Older people’s visit to the ED not rarely mark the beginning of end-of-life (EOL) [7, 8] and the European Resuscitation Council Guidelines 2021 encourages resuscitation shared decision making in emergency care treatment plans [9]. The questions to consider together with older patients in relation to shared decision making is likely to be dependent on the individual patient’s healthcare condition, functional and mental capacity, and the clinical setting in which the questions are framed. Qualitative longitudinal studies have characterized different needs, experiences, and priorities among different groups of older patients approaching the EOL with four dimensions: the physical, psychological, social, and existential/spiritual dimension [10–12]. Importantly, these physical, psychological, social, and spiritual dimensions were described as trajectories of loss and adaptation towards EOL with different patterns according to patients with different functional decline [13]. Having such dimensions in mind might help clinicians to know what to offer and when [13].

We have previously interviewed older Danes about what matters at EOL and what may influence resuscitation preferences [5]. In relation to resuscitation shared decision making, clinicians are encouraged to shift the focus from an isolated cardiopulmonary resuscitation (CPR) decisions to making personalized plans on broader emergency care and treatment [14]. Dimensions of particular importance to be considered by clinicians when CPR decisions are incorporated in such treatment plans are highly relevant knowledge. Investigation of such items structured in dimensions have not been conducted in the ED setting. Hence, we aimed to develop, test, and perform structural validation of items that may influence resuscitation preference among older Danes in the ED setting.

## Methods

This study presents the development, pilot and field-testing, and evaluation of the structural validity of a self-reported questionnaire.

### Development and field-testing of the questionnaire

**Fig 1:**
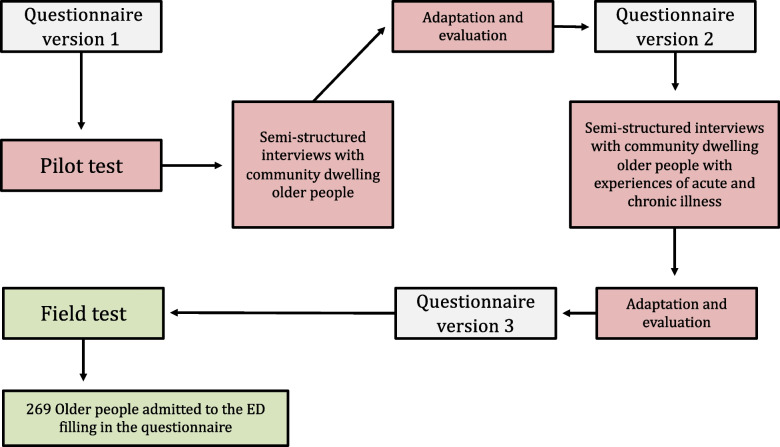
Pilot and field test of items in the questionnaire. The testing phase; qualitative pilot test with two rounds of adaptation and evaluation of items in the questionnaire. In the end, a final version 3 of the questionnaire was ready for a quantitative field test.

To generate items and assess relevant dimensions for resuscitation shared decision making, we developed and field-tested a questionnaire using the six steps described by de Vet et al [15]. Step 1: The construct we intended to measure was defined, i.e., what matters in relation to resuscitation preference for older people. Step 2: Based on existing literature, we conceptualized four dimensions of what matters, previously suggested to be important at EOL; physical, psychological, social, and spiritual [10–13]. Step 3: We used qualitative data from our prior study [5] and performed a theoretical analysis on these data to generate items supported by underlying quotes. We were also inspired by previous literature [16]. Items were selected, pooled in dimensions, and refined by clinical experts during meetings in an ongoing process. Differences in opinion were solved in arbitration meetings. Step 4: A easily understood scale, the 5-point Likert response scale was chosen with 5 representing the “best” score, (e.g., *“Very much”* =5, *“Quite a bit”* =4, *“Somewhat”* =3, *“A little bit”* =2, and *“Not at all”* =1). Responses were allowed to vary depending on the specific item and the completing of these were thoroughly tested by experts and older participants. Step 5: The questionnaire was pilot tested on ten older adults, selected by purposive sampling [17]. Using semi-structured face-to-face interviews, we assessed comprehensibility, completeness, relevance, acceptability, and feasibility of items. We used the techniques “thinking aloud” and “probing” to assess face and content validity [18]. The pilot test consisted of two rounds of interviews; First, with “independent and healthy” community-dwelling older adults, and; Second, with chronic ill older adults with/or without experience of an ED admission. The questionnaire was adapted and evaluated twice between interviews (Fig. 1). Step 6: In the final field-testing step, we investigated internal consistency and structural validity using a cross-sectional study design. All steps are visually presented in an overview in additional file 1.

### Participants and setting

We included patients ≥ 65 years of age admitted to overnight ED stay with a medical condition. Patients were excluded if they did not speak Danish, had known dementia, had a positive Confusion Assessment Method score [19], were treated in the resuscitation bay, or were unable to provide consent. Patients were consecutively sampled during daytime hours on workdays based on admission-list in the last 24-hours. Recruitment took place at four EDs in the Southern Denmark Region between July and November 2019. Patients were informed in written and verbally prior to the completion of the questionnaire both regarding the items included and the question concerning their preference for resuscitation (additional file 2).

### Data collection

Patients filled in the questionnaire (additional file 3), which in the end contained the resuscitation preference question and some demographical questions. Patients rated their own level in physical, psychological, social, and spiritual items. We collected the following demographic information: Age, living arrangement, marital status, educational level, loss of near relative or friend, recollection of any previous EOL conversations with a physician, and if they had an Advanced Care Directive. Resuscitation preference was obtained by the question: “In your current state of health, do you wish that physicians should try to intervene if your heart stops beating?” Responses were divided in four categories: “Cardiopulmonary Resuscitation (CPR)” (those who definitely wanted CPR), “Do not resuscitate” (those who definitely did NOT want CPR), “Uncertain” (which included “I think I want”, “I do not think I want”, and “I’m not sure”), and “I prefer not to answer”.

Research assistants assessed cognitive impairment using the Abbreviated Mental Test Score [20]. We used the Barthel Index to measure the level of dependency in daily living [21] and EuroQoL 5-Dimensions 5-Level index to measure health status and patient reported quality of life [22, 23], see additional file 4.

Patients completed the study questionnaire using an iPad pro 12.9". The setup of the screen was visually adapted for older adults. Data was recorded and stored using REDcap from the Open Patient data Explorative Network at the University of Southern Denmark [24].

### Statistical analysis

Continuous variables were summarized using median and interquartile range (IQR) or mean and standard deviation (SD) as appropriate.

#### Item score-distribution

To ascertain discriminative power of items, distributions of response categories were visualized. A priori, we accepted <3% missing scores and scores of 3-15% were discussed by two authors (SH and JSJ), weighing the importance of the specific item against possible omission. Items with >15% missing responses were discarded as recommended [15].

#### Internal consistency of items, inter-item, and item-total correlation

We evaluated internal consistency by Cronbach’s Alpha and examined inter-item correlation for content overlap within each dimension [15]. An item-total correlation <0.3 was indicative of insufficient correlation, and discrimination ability and deletion were arbitrated.

### Structural Validation

#### Structural validity of conceptualized dimensions

Two models were tested using a three or four-dimensional hypotheses. Model 1 included a physical, psychological, social, and spiritual dimension (factors), derived from step 2 and the theoretical analysis of qualitative data described in step 3. Model 2 consisted of three dimensions (factors), excluding the spiritual dimension and psychological item five (Ps5).

Models were fitted using Confirmatory Factor Analysis (CFA) [25], with weighted least squares mean variation estimation [26]. Fit was assessed by Chi^2^ goodness of fit test, the Root Mean Square Error of Approximation (RMSEA), the Comparative Fit Index (CFI), the Tucker-Lewis Index (TLI), and the Standardized Root Mean square residual (SRMR). Models presented a good fit with the goodness of fit test of larger p-values and RMSEA<0.06, CFI>0.95, TLI>0.95, and SRMR<0.08 [27, 28].

### Post-hoc bifactor analysis

We initially structured our items in four dimensions. However, due to poor fits of the CFA models, a bifactor analysis was chosen to further investigate the dimensionality of the questionnaire [29]. For a measure to validly represent a multidimensional construct, multiple items with heterogenous content needs to be included [30]. The researcher is challenged to balance between trying to measure one thing while simultaneously measuring diverse aspects of this same thing [30]. A bifactor analysis reveals valuable information about item response data that or more or less consistent with both unidimensional (e.g. a strong general factor) and multidimensional (two or more correlated group factors) measurement models [29]. We therefore performed a bifactor analysis post-hoc to assess if the conceptualized multidimensional questionnaire of what matters instead could be unidimensional with an overruling general factor for *what matters*. In the bifactor model, each item was allowed to load on to two factors, the dimension it is assumed to measure and a general factor. It specifies correlations among items that can be accounted for by a general factor [29].

### Measures of dimensionality in the bifactor model

To determine if the bifactor model was appropriate, the strength of the observed dimensionality was assessed using Explained Common Variance (ECV_gen_), which explains the proportion of common variance across items explained by the general factor. Also, biased estimates in factor loadings might be induced when forcing multidimensional data into a unidimensional model was evaluated using the Percent of Uncontaminated Correlations (PUC) [29, 31]. Finally, the percent of Common Variance for a single item (I-ECV) was calculated, this measure indicates what is accounted for by the general factor. All three parameters (ECV, PUC, and I-ECV) were used in combination to determine whether the multidimensional data should instead be a unidimensional model with an overall general factor [31].

### Reliability

Reliability of the bifactor model was assessed by Omega coefficients. Omega accounts for congeneric factor loadings in the model and Omega Hierarchical estimates the proportion of variance in total scores that can be attributed to a single general factor [29].

### Sample size

As a rule of thumb, to obtain robust factor loadings for the structural validity the sample size was aimed to be at least seven participants per item [15].

We used Stata/IC 16.0 (StataCorp. 2019. *Stata Statistical Software: Release 16*. College Station, TX: StataCorp LLC) and MPlus 8.1 [26] to conduct the data analyses.

## Results

### Questionnaire development

A total of 61 items were generated and reduced to 36 after arbitration. Included items are listed in Table 1. The 36-item questionnaire was overall found relevant by 93% of the older participants in the pilot test (additional file 3). Item Ps5 “comfortable with needing help” was rephrased several times due to low comprehension in Danish and translation difficulties. Notably, most participants who did not identify themselves as spiritual or religious in items Sp1-2 “spiritual person” and “religious person” generally misunderstood the meaning of the Danish word for “spiritual” and did not find items Sp3-5 “spiritual or religious beliefs”, “praying”, and “beliefs strengthen when ill” from the spiritual dimension relevant.

**Table 1:** Item distribution of the 36 questions within the physical, psychological, social, and spiritual dimensions of the questionnaire.

**Item number and question**	**Response options**					**Missing n (%)**
**Physical Dimension (P1-P9)**	**Not at all** **n (%)**	**A little bit** **n (%)**	**Somewhat n (%)**	**Quite a bit/lot** **n (%)**	**Very much** **n (%)**	
P1. Getting outside the home	129(48)	40(15)	30(11)	36(13)	34(13)	0
P2. Limitations in dressing or bathing	165(61)	52(19)	25(9)	14(5)	13(5)	0
P3. Limitations in daily activities	92(34)	60(22)	55(20)	44(16)	18(7)	0
P6. Pain	27(10)	53(20)	63(23)	61(23)	65(24)	0
P7. Pain interfere with daily living	61(23)	68(25)	58(22)	53(20)	29(11)	0
P8. Fatigue in general	35(13)	55(20)	62(23)	80(30)	37(14)	0
P9. Fatigue interfere with physical functioning	41(15)	71(26)	58(22)	60(22)	39(15)	0
	**Very much**	**Quite a bit**	**Somewhat**	**A little bit**	**Not at all**	
P4. Managing personal hygiene	186(69)	45(17)	25(9)	11(4)	2(1)	0
	**Excellent**	**Very Good**	**Good**	**Fair**	**Poor**	
P5. Overall physical health	46(17)	86(32)	62(23)	58(22)	17(6)	0
**Psychological Dimension (Ps1-Ps9)**	**Very confident**	**Quite confident**	**Somewhat confident**	**Little confident**	**Not at all confident**	
Ps1. Symptom management at home	49(18)	94(35)	76(28)	36(13)	14(5)	0
Ps2. Humoristic despite symptoms	92(34)	100(37)	54(20)	20(7)	3(1)	0
Ps3. Coping daily living	56(21)	108(40)	62(23)	37(14)	6(2)	0
Ps4. Coping worsening in health	57(21)	110(41)	65(24)	28(10)	9(3)	0
	**Very much**	**Quite a bit**	**Somewhat**	**A little bit**	**Not at all**	
Ps5. Comfortable of needing help	39(15)	69(26)	76(28)	39(15)	46(17)	0
Ps6. Self-control in life	96(36)	95(35)	56(21)	17(6)	5(2)	0
Ps7. Positive about the future	76(28)	87(32)	67(25)	34(13)	5(2)	0
	**Excellent**	**Very Good**	**Good**	**Fair**	**Poor**	
Ps8. Overall mood	52(19)	106(39)	65(24)	37(14)	9(3)	0
Ps9. Overall quality of life	33(12)	90(33)	79(29)	53(20)	14(5)	0
**Social Dimension (1S-9S)**	**Always**	**Usually**	**Sometimes**	**Rarely**	**Never**	
S1. Talk about heath	163(61)	61(23)	28(10)	12(4)	4(1)	1(0.4)
S2. Share worries and fears	158(59)	57(21)	33(12)	13(5)	7(3)	1(0.4)
S3. Support from family	203(75)	33(12)	15(6)	6(2)	11(4)	1(0.4)
S4. Support from others	97(36)	77(29)	55(20)	17(6)	22(8)	1(0.4)
S5. Support from GP	120(45)	102(38)	29(11)	17(6)	0(0)	1(0.4)
S6. Feeling needed	131(49)	77(29)	37(14)	11(4)	12(4)	1(0.4)
	**Not at all**	**A little bit**	**Somewhat**	**Quite a bit**	**Very much**	
S7. Feeling lonely	180(67)	48(18)	22(8)	13(3)	5(2)	1(0.4)
S8. Burden to others	167(62)	66(25)	25(9)	8(3)	2(1)	1(0.4)
S9. Limitations in participating social activities	89(33)	74(28)	47(17)	34(13)	24(9)	1(0.4)
**Spiritual Dimension (Sp1-Sp9)**	**Very much**	**Quite a bit**	**Somewhat**	**A little bit**	**Not at all**	
Sp1 Spiritual person	12(4)	27(10)	37(14)	31(12)	161(60)	1(0.4)
Sp2. Religious person	17(6)	26(10)	46(17)	76(28)	103(38)	1(0.4)
Sp3. Spiritual or religious beliefs	15(6)	33(12)	32(12)	38(14)	72(27)	79(30)
Sp4. Praying	15(6)	19(7)	20(7)	38(14)	98(36)	79(30)
Sp5. Beliefs strengthen when ill	12(4)	12(4)	31(12)	31(12)	104(39)	79(30)
Sp6. Meaning and purpose in life	8(3)	26(10)	81(30)	77(29)	76(28)	1(0.4)
Sp7. Thoughts of death	20(7)	37(14)	85(32)	73(27)	53(20)	1(0.4)
Sp8. Planned funeral	29(11)	27(10)	35(13)	60(22)	116(43)	2(0.7)
	**Perfectly acceptable**	**Acceptable**	**Neutral**	**Unacceptable**	**Totally unacceptable**	
Sp9. Acceptable to talk about death	35(13)	96(36)	110(41)	19(7)	6(2)	3(1)

### Field test

In the field test, 470 older patients were included and 269 (57%) completed the questionnaire. The most common reasons for declining to participate were acute illness (38%) or mental distress (25%). The median age was 76 years (IQR: 71-81), 112 (41%) were women, and 188 (70%) were independent in daily living (Barthel Index≥90) (Table 2). Twenty-four percent did “definitely not” want CPR in case of cardiac arrest in their current health state, whereas 27.7% definitely preferred CPR. Among the older patients, one out of three were uncertain (Table 2).

**Table 2:** Sociodemographic and characteristics of the study population in the field test of the questionnaire (*n*=269).

**Characteristics**	**Study Population n=269**	
**Sex, female, n (%)**	112	(41.6)
**Age, years median (IQR)**	76	(71-81)
**Living Arrangement, n (%)**		
Living with spouse	138	(51.3)
Living with family, friends or other	26	(9.7)
Living alone	105	(39.0)
**Marital Status, (%)**		
Single	14	(5.2)
Married	141	(52.3)
Widowed	65	(24.3)
Divorced/Separated	34	(12.6)
In a relationship	13	(4.9)
Missing	2	(0.7)
**Level of Education, n (%)**		
University degree/Graduate school	16	(6.0)
High school graduate	112	(41.7)
Elementary school or less	133	(49.4)
Did not go to school	4	(1.4)
Missing	4	(1.4)
**Resuscitation preference, n (%)**		
Definitely Yes	74	(27.5)
Uncertain answer	89	(33.1)
Definitely No	63	(23.4)
Prefer not to answer	41	(15.2)
Missing	2	(0.7)
**Loss of friend or near relative, n (%)**		
Yes, loss of spouse	78	(29.0)
Yes, loss of child	31	(11.5)
Yes, loss of friend or other family members	189	(70.3)
No	8	(3.0)
**Barthel Index**		
Median (IQR)	97	(86-100)
<50	13	(4.8)
50-74	22	(8.2)
75-89	45	(16.7)
90-99	66	(24.5)
100	122	(45.4)
Missing	1	(0.4)
**AMTS, n (%)**		
Median (IQR)	10	(9-10)
<7	3	(1.1)
<8	12	(4.5)
<9	21	(7.8)
9-10	230	(85.5)
Missing	3	(1.1)
**EQ-5D-5L index,** median (IQR)	0.745	(0.648-0.838)
Missing	2	
**EQ-VAS,** median (IQR)	55	(45-77)
Missing	2	

*AMTS* Abbreviated Mental Test Score, a score^,^ <9 suggests mild cognitive impairment. Score <8 suggests cognitive impairment. *BI* Barthel Index, score<50: severe dependent in daily living, score 50-74: moderate dependent in daily living, score 75-89: slightly dependent in daily living, score 90-99: minimal dependent in daily living, score: 100, not at all dependent in daily living. *EQ-5D-5L index* EuroQoL 5-Dimensions 5-Level index, ranging from an index of 1.00 indicates full health and 0 (equal to death) to -0.59 (worse state than death). These index values have been converted from population norms.

EQ-VAS score, score 0-100 of overall self-reported current health state. Score 0: the worst health you can imagine, score 100: the best health you can imagine. Numbers are n (%) unless otherwise specified.

### Item score-distribution

Participants used all response categories and most frequently the “best level” on the 5-point Likert response scale. Items Sp3-Sp5 “spiritual or religious beliefs”, “praying”, and “beliefs strengthen when ill” had 79 missing values and accounted for 30% (Table 1). In all other items, less than 1% were missing.

Internal consistency of items, inter-item, and item-total correlation

The internal consistency measured by Cronbach’s Alpha of the four dimensions ranged from 0.70-0.86 (Table 3). We found an inter-item correlation above 0.3, except for Ps5 “comfortable with needing help” and spiritual item 6 (Sp6) “meaning and purpose” (Table 3). Item Ps5 and Sp6 also had the lowest item-total correlations of -0.11 and -0.17, respectively.

**Table 3:** The internal consistency estimate, inter-item, and item-total correlations for each of the four dimensions.

**Dimension**	**No of items**	**Inter-item correlations**	**Item-total correlations**	**Cronbach’s** $\boldsymbol{\alpha }$
Physical	9	0.58-0.79	0.44-0.65	0.86 [0.84;0.89]
Psychological	8	0.64-0.81	0.53-0.73	0.83 [0.80;0.86]
Item Ps5	1	0.09	-0.11	
Social	9	0.33-0.76	0.19-0.66	0.80 [0.76;0.84]
Spiritual	8	0.46-0.79	0.23-0.68	0.70 [0.64;0.76]
Item Sp6	1	0.05	-0.17	

*Item Ps5* Item 5 from the psychological dimension, *Item Sp6* Item 6 from the spiritual dimension.

### Structural validation

#### Confirmatory factor analysis

Model 1 were distributed with nine items loading on each factor for the physical, psychological, social, and spiritual dimension. Overall model fit indexes revealed a poor model fit (Table 4).

**Table 4:** Confirmatory factor analysis and bifactor fit indices for the analyzed models used to evaluate structural validity.

**Analysis**	**Fit Indices**						
	**Chi-2**	**df**	**p**	**RMSEA [90 %CI]**	**CFI**	**TLI**	**SRMR**
**CFA**							
** Model 1:** Four dimensions	1931.6	588	<0.001	0.092 [0.088;0.097]	0.864	0.854	0.112
** Model 2:** Three dimensions	1473.7	296	<0.001	0.122 [0.115;0.128]	0.870	0.858	0.108
**BFM**							
** Model 1:** Four dimensions	991.2	558	<0.001	0.054 [0.048;0.059]	0.956	0.950	0.071
** Model 2:** Three dimensions	562.3	273	<0.001	0.063 [0.055;0.070]	0.968	0.962	0.055

The four dimensions include the physical, psychological, social, and the spiritual dimension. The three dimension has excluded the spiritual dimension.

**Abbreviations:** *BFM* Bifactor Model, *Chi-2* Chi-squared, *CFA* Confirmatory Factor Analysis, *CFI* Comparative Fit Index, *CI* Confidence Interval, *p* p-value *RMSEA* Root Mean Square Error of Approximation, Standardized Root Mean square residual, *TLI* Tucker-Lewis Index.

Model 2 comprised 26 items loading on three factors (physical, psychological, and social dimension). All factor loadings were larger than 0.5 except for item S5 “support from General Practitioner” (factor load of 0.3). Again, the fit indexes revealed a poor model fit (Table 4).

### Post hoc analysis: bifactor model

**Fig 2:**
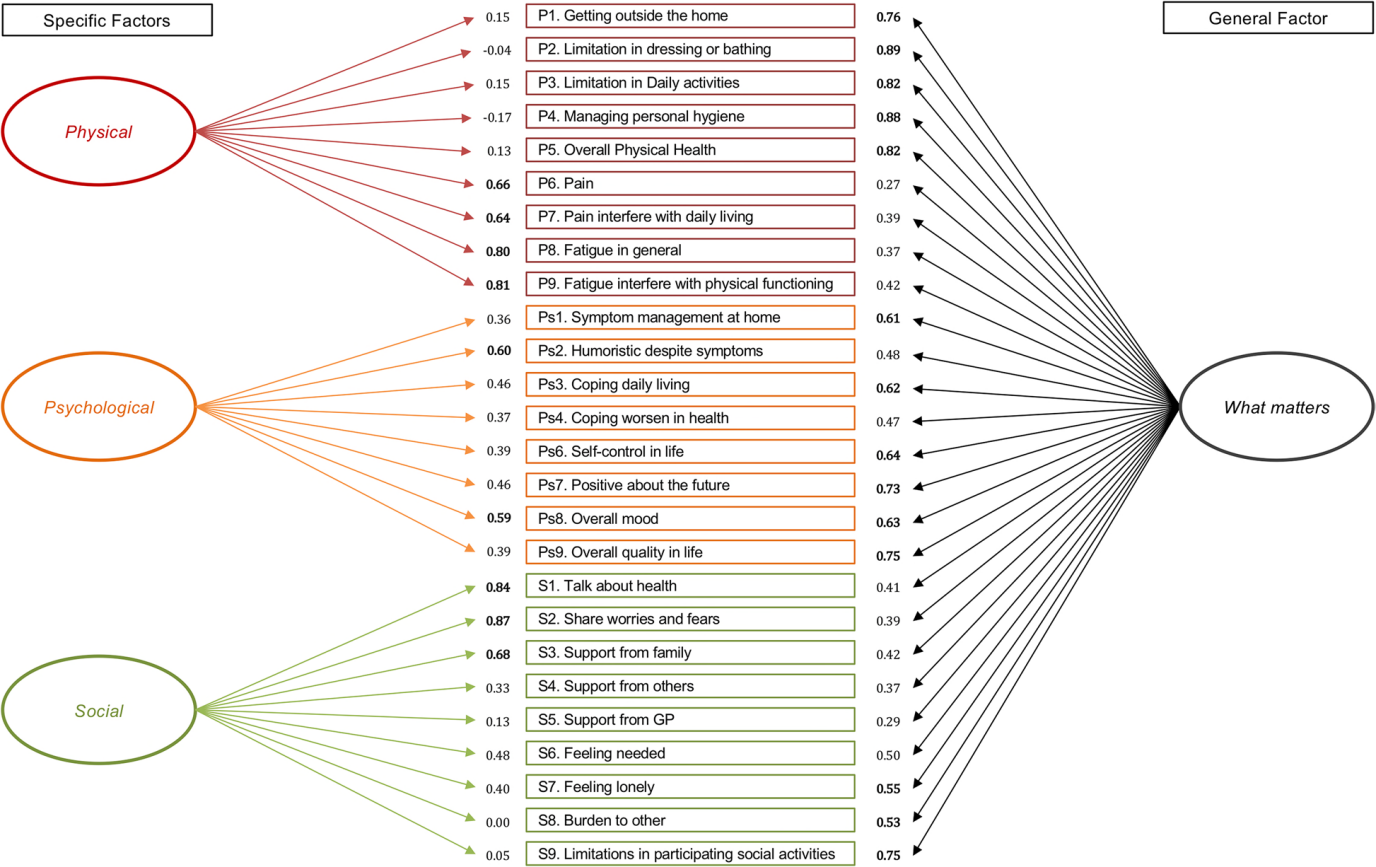
Illustration of the bifactor model with factor loadings. Overall goodness of fit model from the post hoc analysis of the confirmatory factor analysis and bifactor analysis with factor loadings from the three factors and the overall general factor. Factor loadings >0.5 are bolded.

The bifactor analysis on model 1 and 2 revealed information of the dimensionality of the items in the questionnaire and fitted data well. Model 2 excluding the spiritual dimension demonstrated best overall goodness of fit with Chi^2^=562.335 (*p*<0.001), RMSEA=0.063 (90% CI [0.055;0.070]), CFI=0.968, TLI=0.962, and a SRMR=0.055 (Table 4). We found most items were stronger measures of the general factor *what matters* than the specific factors (the dimensions) (Fig. 2.). Items which load stronger onto the general factor *what matters* include activities of daily living (ADL) items, overall self-reported health, symptom management, coping, autonomy, optimism, overall mood, self-reported quality of life, loneliness, burden to other, and social participation in activities.

### Model based reliability analyses

We found high reliability with Omega of 0.96 for the general factor and 0.93, 0.92, and 0.88 for the physical, psychological, and social dimensions, respectively. However, once we accounted for the general factor, the Omega Hierarchical dropped markedly and ranged from 0.19-0.38 for the specific dimensions, together with a high Omega Hierarchical of 0.83 for the general factor. This underscored a strong general factor accounting for the reliable variance in an individuals’ overall responses.

## Discussion

This study is the first to investigate which items to consider in shared decision making for resuscitation preferences in the ED setting. We did not find that the previously identified items where related to four separate dimensions as has been described in palliative care. Items related to the spiritual dimension could be excluded, and the expected multidimensionality of the remaining items could not be confirmed in Confirmatory Factor analyses. Instead, we found the 26 items to be unidimensional with an unknown independent general factor in post-hoc bifactor fit analysis.

Spirituality was a concept difficult to understand and not weighted as important in this Danish ED population. We achieved best acceptable fit without the spiritual dimension in the model and in pilot testing items were misinterpreted by the participants and deemed irrelevant. This is likely explained by a combination of setting and culture. In the UK, where the four dimensions was conceptualized, spirituality was reported important to 70-80% of the general population [32]. In contrast to this, only 14% or 28% of the older patients in the present study considered themselves as spiritual or religious, respectively. This corresponds well with Danes having a cultural attachment to the national church alongside a low personal attachment to the religious practices [33].

We suspect the general factor loading on to all items in the bifactor fit represent independence. The general factor (independence) was a stronger measure of most items compared to the specific factors (dimensions) alone. The items previously deemed important for resuscitation preference did so strongly to items related to living independently. This is unsurprising, as maintaining independence in life is very important for older people [5, 34]. Also, the concept of successful aging have maintained independence in life as a cornerstone [35]. Independence in ADL and greater perceived control of events significantly affected the psychological well-being for older people [36]. Social independence enables participation in social activities and feeling needed, providing a greater sense of purpose in life [37].

Furthermore, items such as coping, self-control in life, optimism, overall mood, and self-reported quality of life (Item Ps1, Ps3, Ps6-9) indicate the importance of being psychological independent in life. Physical, psychological, and social independence might not be equally important to each patient and a distinction between socio-psychological independence and physical independence is necessary [35]. However, the sample population in our study were highly independent in ADL, which may also explain why independence is highly valued and important. Hence, our findings may not be applicable to populations with higher average ADL dependency.

Despite high levels of ADL independence, 24% did definitely not want resuscitation in their current health state (Table 2). This is slightly surprising. However, as stated in the early interviews from an old independently living man: “It’s just a good life that comes to an end” [5]. Evidence is sparse, but older people living independently may not wish to gamble with the outcome of a cardiac arrest if resuscitated [38]. Regardless, resuscitation decision making and goals of care conversations (e.g., Advance Care Planning) needs to be performed before the patients become incapacitated when aiming for fulfillment of patient preference [39].

In the future, clinicians may use this holistic patient-centered questionnaire to identify items that may influence the older person’s resuscitation preference within different settings. Before this final aim can be achieved, further steps involve validation of which items relates to the resuscitation preference. This could be achieved by interviewing patients in shared decision making using the questionnaire prior and during the conversations. For example, decline of a CPR attempt, could be present among independently living older people with good physical health, as found in this present study. In these cases, social or psychological matters may influence one’s resuscitation preference. The final questionnaire items used for quick profiling older peoples influencing factors regarding their resuscitation preference would be further evaluated regarding its responsiveness.

Our study has several strengths. We developed, tested, and validated a questionnaire according to current guidelines [15]. We included a diverse heterogenous sample of older adults and patients in the pilot and field test of the questionnaire to facilitate applicability in both in- and out of hospital settings. We aimed for inclusion of the older ED patients capable of making a resuscitation preference by excluding patients with known dementia, present delirium or confusion as assessed by the treating nurse, and we screened included patients for cognitive impairment. This likely increases the reliability of our results but also limits the applicability to cognitively impaired patients.

This study has limitations, which might limit the generalizability of our findings to other EDs or healthcare settings. Firstly, one important implication of this study is the limited transportability to English-speaking populations which would require cultural adaptation and translation, but that is beyond the scope of this study. Secondly, the structural validation with CFA and bifactor analyses would have benefitted from a larger sample size [40]. Thirdly, cognitive interviews at different ED sites would have strengthened the content validity of the questionnaire. However, items were generated from in-depth interviews with older ED patients and the pilot test included older adults previously admitted to the ED. Fourthly, we aimed at including patients from four EDs but primarily tested the questionnaire in one ED (84.4% of the patients included), which also limits the transportability of our findings. However, our findings highlight the importance of understanding which items that may be valued and how items may be structured in dimensions in relation to shared decision making, regardless of setting. Finally, we cannot conclude whether items relate to how the older patient feel about resuscitation, as the linkage of how the general factor relate to the older patients’ willingness to accept resuscitation was not studied. Importantly, end-of-life factors that matter most were asked at the same time as their resuscitation preferences, yet what matters most in regard to end-of-life decision making remains unknown. However, this study provides a novel insight into which items may be important to consider in conversations with older mostly independent patients in the ED, as this might influence their resuscitation preference. To develop a full picture, additional studies are needed in other clinical settings, such as the intensive care unit and out-of-hospital settings.

## Conclusion

We developed a questionnaire to investigate the dimensionality of what matters and may influence resuscitation preferences among older ED patients. The hypothesized 36 items in four dimensions were not confirmed. In a post-hoc bifactor analysis items were found to be reliable as a unidimensional 26-item measure of a general factor, which may represent the overall construct of independence. Pending further testing and validation of the relation between items and one’s resuscitation preference, these findings should be used to increase awareness of addressing different matters related to independence, when discussion CPR, completion of end of life forms, emergency treatment care plans, or advance care directives.

## Supplementary Information

**Additional file 1.** Supplementary file

## Data Availability

The datasets used and/or analysed during the current study are available from the corresponding author on reasonable request.

## Abbreviations

ADL: Activities of daily living
CFA: Confirmatory Factor Analysis
CFI: Comparative Fit Index
CPR: Cardiopulmonary Resuscitation
ECV: Explained Common Variance
ED: Emergency Department
EOL: End-of-life
IQR: Interquartile Range
I-ECV: Percent of Common Variance for a single item
PUC: Percent of Uncontaminated Correlations
Ps5: Psychological item 5
RMSEA: Root Mean Square Error of Approximation
SD: Standard Deviation
SRMR: Standardized Root Mean square residual
TLI: Tucker-Lewis Index
