# Supplementary material for: What matters and influence resuscitation preference? Development, field testing, and structural validation of items among older patients in the emergency department
Source: BMC Geriatr. 2022 Dec 23;22:995. doi: 10.1186/s12877-022-03707-y (PMC9783719; doi:10.1186/s12877-022-03707-y)
Supplement: Supplementary file 1 — Additional file 1. Supplementary file [file 12877_2022_3707_MOESM1_ESM.docx]

# Supplementary File (additional file 1-4)

### **Additional file 1**

### **Title:** The Methodological process

### **Description:** The methodological process and considerations in questionnaire development.

### **Additional file 2**

### **Title:** Oral and written introduction to the questionnaire

### **Description:** The information given to participants before completion of the questionnaire.

### **Additional file 3**

### **Title:** English questionnaire

### **Description:** The final version of the English questionnaire used in the field-test.

### **Additional file 4**

### **Title:** Instruments and tests

### **Description:** A detailed description of the instruments used in the field-test.

# Additional file 1; The methodological process

**Figure:** The methodological process and considerations in questionnaire development. The left column represents the steps moving from research questions to major themes, to the theoretical framework to inclusion of keywords, to items generation of the intended end-product of the questionnaire. The mid column represents the products of the left column. The right column represents the methods used or where the information was gathered from to achieve the product/results.

# Additional file 2; Oral and written introduction to the questionnaire

**Oral information (translated into English):**

First of all, thank you for being interested in this study.

The overall purpose of this study is to improve the quality of treatment in patients aged 65 years or above. We will aim to do so by knowing you as well as knowing your attitude towards treatment. Therefore, we ask you to fill in this questionnaire.

The questionnaire is targeted people aged 65 years or above. Older people of your age have shaped the content of the questions by indicating what thoughts they have about the last part of life, cardiopulmonary resuscitation in case of cardiac arrest including what is important in the last part of life.

In the end of the questionnaire, we would like to ask you about your preference for cardiopulmonary resuscitation in the event of a cardiac arrest. Here you can always choose not to answer if you have not thought about this. The other questions may help us understand your preference for cardiopulmonary resuscitation and give us indications of needs you may have we were not aware of.

**Written information directly copied from the database (In Danish):**

From RedCap OPEN In Danish:

”Kære [baseline_arm_1][stam_name]

Først og fremmest, tak fordi du vil udfylde dette spørgeskema.

Formålet med dette spørgeskema er at forbedre kvaliteten af behandlingen hos ældre der nærmer sig den sidste del af deres naturlige liv. Vi vil gerne lære om dig og dine livserfaringer. Dine svar kan give os en indikation om, hvilke behov der i hverdagen er hos dig. I slutningen af spørgeskemaet vil vi gerne spørge dig om dit ønske til forsøg af genoplivning i tilfælde af at du skulle få et hjertestop. Dette vil hjælpe os med at forstå om du har ønske til behandling eller fravalg af behandling som vi ikke kendte til.

Ældre på din alder har formet indholdet i spørgsmålene, ved at tilkendegive hvilke tanker de gør sig omkring den sidste del af livet, herunder hvad der for dem er livskvalitet og vigtigt i hverdagen.

Det er helt FRIVLLIGT at deltage. Du må gerne tale med dine pårørende eller en ven, inden du beslutter dig. Din beslutning vil ikke få konsekvenser for din fremtidige behandling, pleje eller anden kontakt til sundhedsvæsenet.

Undersøgelsens resultat vil blive brugt i et større projekt. Dine tanker og holdninger vil være med til at skabe indholdet i mindst én videnskabelig artikel. I fremtiden kan du sætte dit præg på, hvilke behov vi skal være opmærksomme på i forbindelse med ældre patienters indlæggelse via akutmodtagelsen.

Derfor vil vi igen sige MANGE TAK for din hjælp med at udfylde dette spørgeskema!

Dette projekt er under udvikling og derfor vil svarene kun bruges som et forsøg, og IKKE deles med personalet her på afdelingen eller hospitalet, ej heller til din praktiserende læge.

**Hvis du skulle have et ønske om, at dit plejepersonale eller læge skal kende til dine ønsker eller behov, skal du selv oplyse dem om disse.”**

**Written information from the database (translated into English):**

"Dear [baseline_arm_1][stam_name]

First of all, thank you for completing this questionnaire.

The purpose of this questionnaire is to improve the quality of treatment in elderly people approaching the last part of their natural life. We want to learn about you and your life experiences. Your answers can give us an indication of what needs you have in everyday life. At the end of the questionnaire, we would like to ask you about your wish to attempt resuscitation in the event of a cardiac arrest. This will help us understand whether you have a desire for treatment or decline of treatment that we were not aware of.

Older people of your age have shaped the content of the questions by indicating what thoughts they have about the last part of life, including what for them is quality of life and important in everyday life.

It is completely VOLUNTARY to participate. You may want to talk to your relatives or a friend before you decide. Your decision will not have consequences for your future treatment, care or other contact with the healthcare system.

The results of the study will be used in a larger project. Your thoughts and opinions will help to create the content of at least one scientific article. In the future, you can leave your mark on which needs we must be aware of regarding the admission of elderly patients via the emergency department.

Therefore, we would like to say THANK YOU again for your help filling in this questionnaire!

This project is under development and therefore the answers will only be used as an experiment, and will NOT be shared with the staff here at the ward or hospital, nor with your GP.

If you should have a wish for your care staff or doctor to know about your wishes or needs, you must inform them about these yourself.”

# Additional file 3; English questionnaire

The questionnaire in the final English version. The grey rectangles represent “new page”on the Ipad.

P1. Does your health now limit you in going OUTSIDE the home, for example to shop or visit a doctor’s office?

Not at all A little bit Somewhat Quite a lot Very much

    

P2. Does your health now limit you in bathing or dressing yourself?

Not at all A little bit Somewhat Quite a lot Very much

    

P3. Does your physical health limit you in performing your daily activities?

Not at all A little bit Somewhat Quite a lot Very much

    

P4. Can you manage your own personal hygiene?

Not at all A little bit Somewhat Quite a bit Very much

    

P5. In general, how would you rate your overall physical health?

Excellent Very good Good Fair Poor

    

**In the following questions please think of the past 7 days**

P6. Did you experience pain or discomfort?

Not at all A little bit Somewhat Quite a bit Very much

    

P7. How much did pain interfere with your enjoyment of life?

Not at all A little bit Somewhat Quite a bit Very much

    

P8. How fatigued were you on average?

Not at all A little bit Somewhat Quite a bit Very much

    

P9. To what degree did your fatigue interfere with your physical functioning?

Not at all A little bit Somewhat Quite a bit Very much

    

**Please indicate your current level of confidence in yourself managing the following statements:**

Ps1. “I can manage my symptoms when I am at home”

I am I am I am I am I am

very quite somewhat a little not at all

confident confident confident confident confident

    

Ps2. “I can maintain my sense of humor despite my symptoms”

I am I am I am I am I am

very quite somewhat a little not at all

confident confident confident confident confident

    

Ps3. “I can find new ways to manage daily activities when the old way doesn’t work”

I am I am I am I am I am

very quite somewhat a little not at all

confident confident confident confident confident

    

Ps4. “I can cope well if my health is deteriorating” (e.g. an admission to hospital)

I am I am I am I am I am

very quite somewhat a little not at all

confident confident confident confident confident

    

Ps5. How comfortable are you with needing help (or the thought of needing help) from others in daily living such as bathing, dressing or feeding yourself?

Very much Quite a bit Somewhat A little bit Not at all

    

Ps6. How much do you feel that you are in control of your life?

Very much Quite a bit Somewhat A little bit Not at all

    

Ps7. How positive do you feel about the future?

Very much Quite a bit Somewhat A little bit Not at all

    

Ps8. In general, how would you rate your overall mood?

Excellent Very good Good Fair Poor

    

Ps9. In general, would you say your quality of life is………..

Excellent Very good Good Fair Poor

    

S1. I have people who I can talk to about my health…………

Always Usually Sometimes Rarely Never

    

S2. I have someone with whom to share my most private worries and fears……….

Always Usually Sometimes Rarely Never

    

S3. I get emotional support from my family members……….

Always Usually Sometimes Rarely Never

    

S4. I get emotional support from others than my family e.g. friend, neighbor etc.

Always Usually Sometimes Rarely Never

    

S5. I generally feel supported and understood by my general practitioner………..

Always Usually Sometimes Rarely Never

    

S6. I have someone who makes me feel needed……….

Always Usually Sometimes Rarely Never

    

S7. Do you feel lonely?

Not at all A little bit Somewhat Quite a bit Very much

    

S8. Do you feel like a burden to others?

Not at all A little bit Somewhat Quite a bit Very much

    

S9. Is your health preventing you from participating in social activities?

Not at all A little bit Somewhat Quite a bit Very much

    

Sp1. Are you a spiritual person?

Very much Quite a bit Somewhat A little bit Not at all

    

Sp2. Do you consider yourself a religious person?

Very much Quite a bit Somewhat A little bit Not at all

    

Sp3. Do you find comfort in your faith or spiritual beliefs?

Very much Quite a bit Somewhat A little bit Not at all

    

Sp4. How much do you pray?

Very much Quite a bit Somewhat A little bit Not at all

    

Sp5. During times of illness, have your religious or spiritual beliefs been strengthened?

Very much Quite a bit Somewhat A little bit Not at all

    

Sp6. Are your meaning and purpose in life challenged by your illness or decline in your health?

Very much Quite a bit Somewhat A little bit Not at all

    

Sp7. Do you worry about dying?

Very much Quite a bit Somewhat A little bit Not at all

    

Sp8. To what extent have you planned your funeral?

Very much Quite a bit Somewhat A little bit Not at all

    

Sp9. How acceptable is it in your environment to talk about death and dying?

Perfectly acceptable Acceptable Neutral Unacceptable Totally unacceptable

    

D1. Which of the following describes best your living arrangement?

(Tick all relevant boxes)

Living with spouse 

Living with adult children 

Living with your family 

Living with friends 

Living with older people (e.g. a nursing home or aged care facility) 

Living alone 

D2. Which of the following describes your marital status? (Tick only 1 box)

Single, never married 

Married 

Widowed 

Widowed and married again 

Divorced/Separated 

In a relationship 

D3. Which of the following describes best your highest level of education? (Tick only 1 box)

I completed graduate school 

I graduated from college/High School 

I finished primary school 

I did not go to school 

D4. Have your doctor ever diagnosed you with any of the following conditions, such as:

Please tick all that apply

Heart attack or cardiovasculare disease Yes 

Cancer Yes 

Arthritis Yes 

Stroke Yes 

Liver disease Yes 

Kidney disease Yes 

Heart faillure Yes 

Lung disease Yes 

Metabolic disease Yes 

None of above 

D5. Have you ever had an event that affected your physical function?

(E.g. a fall, a stroke, or cause?)

Yes 

No 

D6. Have you previously experienced a loss of a relative or a dear friend?

Yes, I lost my wife/husband 

Yes, I lost my child 

Yes, I lost a family member 

Yes, I lost a friend 

No, I never lost someone close to me 

E1. Have you previously been asked by a physician about your wish regarding resuscitation if your heart stop beating?

Yes 

No 

I do not remember 

E2. In your current state of health, do you wish that physicians should try to intervene if your heart stops beating?

I definitely want them to 

I think I want them to 

I’m not sure 

I do NOT think I want them to 

I definitely do NOT want them to 

I prefer not to answer this question 

E3. Have you completed written forms regarding your wishes at the end of life?

(e.g. a living will, a care plan, “my last will” etc.)

Living will 

Advance Directive 

“My last will” 

Advance Care Plan 

Another formula than mentioned above 

I do not remember 

None of above 

E4. Did anyone help you fill in this questionnaire?

Yes, I have got help to much of it 

Yes, I have got help to some questions 

No, I filled it in by myself 

E5. How relevant did you find this questionnaire to be?

Very relevant Relevant Neutral Slightly relevant Not relevant

    

# Additional file 4; Instruments and tests

# A detailed description of the instruments used in the field-test

**Confusion Assessment Method (CAM)**^19^**:**

We used the CAM test to screen for delirium and excluded patients with a positive test. It consists of four main criteria and in various modified versions. CAM-ICU is translated and validated for use in Denmark. A systematic review confirms it can be used as a screening tool for delirium internationally^40^.

**Abbreviated Mental Test Score (AMTS)**^20^**:**

We used the AMTS to assess for cognitive impairment among the older patients. The 10-question AMTS was recommended as a brief pragmatic test of cognitive function and found applicable to the majority of older patients with an emergency admission^41^. A total score of 10 can be achieved as maximum indicating cognitive well-functioning. We used the cut-off score <7, as this was found to be an accurate test of cognitive impairment^42^.

**The Barthel Index**^21^**:**

We scored patients at the time of inclusion with Barthel Index to get information of limitations in activities of daily living (ADL). A standardized validated scale describes the patient’s ability to perform simple tasks within ten items of ADL, involving feeding, grooming, bathing, dressing, control over bowel and bladder along with mobility. Total score ranges from a score of 0 (total dependent) to a maximum of 100 (totally independent).

**The EuroQol5Dimensions-5Levels (EQ-5D-5L)**^22,23^**:**

We used the simple generic instrument EQ-5D-5L to measure health status of the patients. This descriptive system comprises 5 dimensions: mobility, self-care, usual activities, pain/discomfort, and anxiety/depression. Each dimension has 5 levels ranging from no problems to extreme problems. The EQ-5D index score ranging from below 0 (where 0 is the value of a health state equivalent to dead; negative values representing values as worse than dead) to 1 (the value of full health), these can be converted from the population norms^43^.

A visual analogue scale (EQ-VAS) records the patient’s self-rated health on a scale from 0 (the worst health you can imagine) to 100 (the best health you can imagine). The EQ-5D-5L and EQ-VAS are reliable, valid, and responsive in a wide range of conditions and populations.
